# Emergency Remedies

**Published:** 1910-08

**Authors:** George L. Servoss

**Affiliations:** Fairview, Nevada


### TEXAS MEDICAL JOURNAL.

Established July, 1885

F. E. DANIEL, M. D., - - - - - - Editor, Publisher and Proprietor

Published Monthly.—Subscription $1.00 a Year.

VOL. XXVI AUSTIN, AUGUST, 1910. No. 2

The publisher is not responsible for the views of the contributors.

### Original Articles.

For Texas Medical Journal.

### Emergency Remedies.

BY GEORGE L. SERVOSS, M. D., FAIRVIEW, NEVADA.

While there are hundreds of remedial agents offered for the relief and cure of diseased conditions, it has been found by the majority of physicians that only a few of the many are absolutely necessary. In fact, it has been shown that the average man familiarizes himself with only a few and employs them in the treatment of all conditions. Some have said that they could get along with five agents, and practice successfully, but that is undoubtedly drawing the line too fine, but with the twenty-four agents mentioned hereafter the physician will be able to cope with any symptoms appearing before his notice, and in the majority of instances will be able to bring about happy terminations without going outside this list.

1. *Aconitine.*—There are two alkaloids, the crystalline and amorphous, obtained from the leaves and roots of the Aconitum napellus, but only the latter will be considered at this time, as the former is too potent for employment as a remedial agent.

Aconitine amorphous is a marked antipyretic, as well as a very reliable analgesic. It acts by slowing the heart, lowering the blood pressure and equalizing the circulation.

Aconitine may be employed alone, or in combination with digitalin gr. 1-67 and strychnine arsenate gr. 1-134, or with digitalin gr. 1-67 and veratrine gr. 1-134, as an antipyretic. The dosage of aconitine, either alone or in the above mentioned combinations, is 1-134 gr. repeated at intervals of from fifteen minutes to one-half hour, until such time as the effect is shown by falling of the temperature and pulse.

2. *Acid Arsenous.*—While this is not an emergency remedy, per se, it is one which is worthy of much larger employment than is usually given it. Arsenous acid has marked tonic alterative properties. Its uses and applications are too well known to require description. The dose recommended is 1-67 gr. at intervals of from one to two hours to effect.

3. *Aloin.*—Aloin is a very reliable laxative and cathartic. It acts by stimulating the liver and irritation of the colon. In minimum doses it is a stomachic and tonic, while large, full doses are emmenagog. The actions and dose are so well known as to require very little attention. Aloin may be administered alone, but is more frequently used in laxative combinations, of which a considerable number have been suggested. The dose suggested is from gr. 1-12 to gr. 1-6.

4. *Atropine Sulphate.*—It is very probable that we have no emergency remedy of greater importance than is atropine. It has a multitude of applications, and, while it is a drug to be employed with care, it is one of the greatest life savers with which the doctor is armed. In collapse, with clammy skin, contracted pupils and other symptoms common to this condition, atropine in full dosage, hypodermically, will bring about an equalization of the circulation and immediately relieve localized congestions. It relieves hemorrhages, especially those of an internal nature, by attracting the flow of blood to the surface. It also has a marked analgesic action and may be employed instead of morphine and other opium products in many cases. Atropine, used early in congestive processes of the respiratory tract, will not infrequently, act as an abortifacient. While atropine is a well and favorably known remedy, it is one which is worthy of constant study and application, especially in emergencies. The full dose is 1-100 gr. and this is usually employed in a single dose, hypodermically, but for oral exhibition, where the drug is to be employed for some time, the suggested dosage is from 1-1000 gr. to 1-250 gr. Care must be taken in all instances in regard to the dosage, in that the amount employed be just enough and no more, as this is a very potent remedy and the matter of overdosage is of great importance.

5. *Brucine.*—Brucine is the sister alkaloid of strychnine, in nux vomica, and has much the same action as has the latter, but is milder and may be administered in larger doses. It is suggested in cases where the slighter effects are desired. It is a general tonic and is indicated in all instances where a remedy of that sort should be employed. Brucine is preferable to strychnine in the treatment of children. The dose is 1-134 gr. at such intervals as may be indicated, although in some marked conditions, where prompt action is required, this dosage may be increased to a considerable extent.

6. *Calcium Sulphide.*—In calcium sulphide we find another markedly important emergency remedy. To combat infectious processes, it is probable that we have no other remedy of like importance. It either inhibits the action of, or destroys, bacteria of all sorts. It has been found that it abates the fury of all eruptive diseases and has much to do with prompt recovery from smallpox. In the latter disease, when the sulphide is employed to full effect, there is less tendency to pus formation and consequent disfigurement. Calcium sulphide will abort the course of all purulent conditions. It is claimed that a person saturated with this agent is amune to the contraction of contagious diseases. Not only is this remedy of service in eruptive diseases, but in all other conditions due to ineffective invasion. The dose is from 1-6 gr. to 1-2 gr. and should be administered until such time as there is a full saturation, this being denoted by the appearance of sulphurous odors in all of the natural secretions.

7. *Calomel.*—Calomel is so well, and favorably, known, as not to require any extended discussion. It is the cathartic and laxative par excellence. It is one of the most reliable eliminants offered, as it not only acts as an hepatic stimulant, but has marked diuretic powers, and serves a double purpose in overcoming toxemias of all sorts. There is much discussion regarding the proper dosage of this agent, but the writer prefers administering it in 1-6 gr. doses at half-hourly intervals until one grain has been given. It has been found that podophyllin acts as a synergist to the action of calomel, and I have found it good routine to employ a like amount of that principle with the calomel.

8. *Camphor Monobromated.*—This agent may be employed wherever the bromides are indicated and acts as an antispasmodic and hypnotic. It is especially indicated where there is hyperesthesia of the genital tract. The camphor content of this salt acts to prevent the depression which so frequently follows the exhibition of the alkaline bromides. This is a drug which is worthy of more attention than has been given it in the past. The standard dose is 1-12 gr. given to effect.

9. *Codeine.*—Codeine has all the good effects, with few of the bad ones of its sister alkaloid of opium, morphine, and is indicated in the majority of instances where the latter salt would be employed. The action of this drug is well and favorably known and needs no discussion at this time. It will be found to be a reliable analgesic, antispasmodic and hypnotic, and to relieve spasmodic cough. It should be employed where opiates of the morphine class are indicated. The dose is from 1-67 gr. to 1-6 gr. of the sulphate salt.

10. *Colchicine.*—In certain conditions, colchicine is one of the best eliminants we have. It stimulates and increases the secretions of the liver, kidneys, intestinal glands and the skin, and in this way is valuable to relieve toxemias of all varieties, and more especially those of a gouty, rheumatic or autoinfective nature, wherein the blood and tissues are saturated with effete material. In conditions of this sort, where there is an elevation of the temperature and blood pressure, it acts as a valuable synergist to the antipyretics. Care should be taken in the exhibition of this remedy to carry it to therapeutic effect and no further, for, if carried beyond that point, vomiting and purging are liable to follow. From the above, the indications are apparent. For slow effect the dose is 1-134 gr. three or four times a day, but if quick action is desired the intervals should be of greater frequency. This is an agent worthy of great study and frequent application, especially as an emergency remedy.

11. *Copper Arsenate.*—It is only within recent years that the value of this remedy in the treatment of duodenal derangements has been recognized. It has been found very effective, either alone or in combination with the sulphocarbolates, in the treatment of cholera infantum, and is considered as a specific in that condition. It is indicated in many of the bowel disturbances peculiar to the heated summer months. As an emergency remedy, this salt should be given close attention. The dose recommended is from 1-1000 gr. to 1-250 gr. given at frequent intervals to effect.

12. *Digitalin.*—Digitalin is the active principle of the whole drug digitalis and has all of the action of the entire plant. While digitalis products are all slow of action, as a rule, this glucoside gives reaction in a comparativley short time, usually within a half-hour to an hour. This principle is eliminated rapidly and has no cumulative action, common to and feared from preparations of this drug. Like the entire drug, digitalin is a heart tonic and diuretic, and is indicated in all cases where digitalis is used. It has been found very effective in combination with aconitine for the relief of high temperatures, especially in asthenic cases. The dose recommended is 1-67 gr. repeated at frequent intervals to obtain rapid results, or three times a day where prolonged action is desired.

13. *Emetine.*—Emetine is one of the active principles of ipecac and has all of the good actions of that drug, without its bad effects, which latter are due to the actively emetic principle—cephaelin. Emetine, like ipecac, is a very valuable expectorant and is indicated in cases where there is a lack of secretion. It also increases the secretions of the intestinal tract and stimulates the liver function. It is mildly emetic, and, by giving full doses, emesis may be obtained, but it is not employed in this manner, as a rule, although in emergency may be used in this way, providing no other emetic is at hand. The more prominent indications are cough with little, or no, secretion and in certain bowel disorders, particularly dysentery. It is very useful to stimulate the secretions in pneumonia and bronchitis. The dose as an expectorant is 1-67 gr. to 4-67 gr. at intervals sufficiently frequent to obtain results desired. For marked stimulation of the liver function, or to produce emesis, as high as 1 gr. doses, single, may be given.

14. *Ergotin.*—Ergotin is a purified aqueous extract of ergot, and carries all of the properties of that drug. It stimulates non-voluntary muscles, but is somewhat less oxytocic in action than is the fluid extract of the drug. One gr. equals in action about one-half dram of the standard fluid extract. Ergot in this form is much pleasanter of administration and the action derived is more satisfactory. The dose is to be regulated according to the conditions present.

15. *Glonoin.*—In glonoin, or nitroglycerine, we have one of our most reliable and effective remedies. Its action is very rapid and it is indicated in syncope, heart failure, congestion with chills, and in all other instances where there is an acute error in the circulation, with internal congestions. It acts by flushing the capillaries and carrying the blood with rapidity from the congested areas. Its action is prolonged by the exhibition of atropine, hyoscyamine and strychnine in combination with it. Glonoin gives its activity prior to reaching the stomach; in fact, seems to be more active when absorbed from the buccal mucous membranes than from the gastric. Glonoin should be in the hypodermic case of every physician, in that it may be at hand at a moment's notice. The dose ranges from 1-250 gr. to 1-100 gr. Glonoin acts as a valuable synergist to all of the antispasmodics.

16. *Hyoscyamine Amorphous.*—This is an alkaloid from Hyoscyamus niger and is an antispasmodic and relaxant. It is probably the best antispasmodic we have and in addition is a very good hypnotic. It has been used with good effect, in combination with strychnine in the relief of intestinal colic and strangulated hernia. Space will not permit of giving all of the indications of this valuable remedy. The dose is 1-250 gr. in adults, to be given at intervals of from fifteen to thirty minutes until relief or dilatation of the pupils and then at longer intervals to sustain the effect. Strychnine and glonoin are synergists to that action of hyoscyamine.

17. *Morphine Sulphate.*—Morphine is too well known, as to action, application and dosage, as to need other than passing notice that it is a valuable emergency remedy in certain conditions and should always be at hand.

18. *Podophyllin.*—Like calomel and many others of the commoner drugs, podophyllin is well known. It is a very valuable remedy in emergencies where a reliable cathartic is required and acts well in combination with calomel. The dose suggested is the same as of calomel, 1-6 gr. every half hour, until 1 gr. has been exhibited. Followed by a saline, there is an assurance that the alimentary canal has been thoroughly cleansed.

19. *Lithium Benzoate.*—While there is considerable discussion regarding the efficacy of the lithium salts as a whole, it is a recognized fact that the benzoate salt stimulates the action of the kidneys to increased elimination with increased flow of urine and sedation of the urinary tract. It also has an antiseptic action upon this tract, with rapid correction of purulent conditions. The particular indication is in catarrh of the bladder, and gives relief, in combination with hyoscyamine, in painful micturition. The dose is from 1-6 gr. to 1-2 gr. with copious draughts of water at intervals of from one to two hours until the urine is rendered alkaline. This agent is used in combination with colchicine and other eliminants and meets many indications not mentioned at this time.

20. *Quinine Arsenate.*—The actions and uses of quinine are well known and it would be a waste of time to go into detail regarding them. The arsenate salt recommends itself as superior to other salts, in that we here have the combined tonic actions of quinine and arsenic.

21. *Quassin.*—Quassin is the active principle of quassia, and is a very valuable tonic to the digestive functions, especially of the buccal cavity and the stomach. It also increases the flow of bile. It is one of the best and most reliable stomachics we have and acts to increase the appetite in the same manner as do other bitters. It is indicated in atonic and catarrhal conditions of the digestive tract, in which conditions it is well to combine strychnine with quassin. The dose is from 1-67 to 4-67 gr. before meals.

22. *Strychnine Arsenate.*—The remarks made regarding quinine arsenate are applicable to the arsenate salt of strychnine, in that we have the combined tonic action of the arsenic and strychnine. The indications of this salt of strychnine are the same as others, all of which are well and favorably known.

23. *Veratrine.*—Like aconitine, veratrine has marked antipyretic actions. It lowers temperature by slowing the heart and dilating the capillaries, thereby equalizing the circulation. It also stimulates all of the eliminating functions and is indicated, because of this, in infective processes where considerable amounts of toxins are stored up. The specific indications are uremia and eclampsia, although it may be employed with good effect in many other pyrexias, either alone or in combination with aconitine and digitalin. Veratrine is a gastric irritant and should be given in connection with plenty of fluid. The indication of full effect, other than reduction of the pulse and temperature, is a sense of gastric warmth and the remedy should be discontinued should this symptom appear. The dose is 1-134 gr. at half-hour to hourly intervals to effect.

24. *Zinc Sulphocarbolates.*—With the recognition of autotoxemia and the relation it bears to other conditions, we are bound to see the value of intestinal antiseptics, and experience has shown that the sulphocarbolates are very effective and that they may be employed to full effect without fear of any untoward secondary action. The zinc salt combines the antiseptic action with that of an astringent and is indicated in every case where it has been determined that there is an infective condition of the alimentary tract. The other sulphocarbolates are those of sodium and calcium, which act as antiseptics, without astringent action. The calcium salt is indicated in those cases where there is an apparent lack of the lime salts and the soda salt when simple intestinal antisepsis is desired. The three salts are frequently employed in combination, but, in emergencies, it will be found that the zinc salt will bring about the results desired. The dose ranges from 1-6 gr. to as high as 5 grs. and, if a pure salt is employed, there need be no fear regarding any untoward results accruing from the use of the larger dose.

While we have classed the above as emergency remedies, the observant physician will find, with this equipment, that he will be able to meet the requirements in the average case coming under his observation. To the above list I would add a good saline laxative and cathartic and possibly a few other drugs for use in other than emergency cases, but believe that, if this list is studied closely, it will be found that the items mentioned will meet about all the indications met with in an everyday practice.

